# Novel functional insights into the microbiome inhabiting marine plastic debris: critical considerations to counteract the challenges of thin biofilms using multi-omics and comparative metaproteomics

**DOI:** 10.1186/s40168-024-01751-x

**Published:** 2024-02-22

**Authors:** Lauren F. Messer, Charlotte E. Lee, Ruddy Wattiez, Sabine Matallana-Surget

**Affiliations:** 1 Division of Biological and Environmental Sciences, Faculty of Natural Sciences, University of Stirling, Stirling, FK9 4LA Scotland; 2 Proteomics and Microbiology Department, University of Mons, Mons, 7000 Belgium

**Keywords:** Marine plastisphere, Microbial community function, Multi-omics, Comparative metaproteomics

## Abstract

**Background:**

Microbial functioning on marine plastic surfaces has been poorly documented, especially within cold climates where temperature likely impacts microbial activity and the presence of hydrocarbonoclastic microorganisms. To date, only two studies have used metaproteomics to unravel microbial genotype–phenotype linkages in the marine ‘plastisphere’, and these have revealed the dominance of photosynthetic microorganisms within warm climates. Advancing the functional representation of the marine plastisphere is vital for the development of specific databases cataloging the functional diversity of the associated microorganisms and their peptide and protein sequences, to fuel biotechnological discoveries. Here, we provide a comprehensive assessment for plastisphere metaproteomics, using multi-omics and data mining on thin plastic biofilms to provide unique insights into plastisphere metabolism. Our robust experimental design assessed DNA/protein co-extraction and cell lysis strategies, proteomics workflows, and diverse protein search databases, to resolve the active plastisphere taxa and their expressed functions from an understudied cold environment.

**Results:**

For the first time, we demonstrate the predominance and activity of hydrocarbonoclastic genera (*Psychrobacter*, *Flavobacterium*, *Pseudomonas*) within a primarily heterotrophic plastisphere. Correspondingly, oxidative phosphorylation, the citrate cycle, and carbohydrate metabolism were the dominant pathways expressed. Quorum sensing and toxin-associated proteins of *Streptomyces* were indicative of inter-community interactions. Stress response proteins expressed by *Psychrobacter*, *Planococcus*, and *Pseudoalteromonas* and proteins mediating xenobiotics degradation in *Psychrobacter* and *Pseudoalteromonas* suggested phenotypic adaptations to the toxic chemical microenvironment of the plastisphere. Interestingly, a targeted search strategy identified plastic biodegradation enzymes, including polyamidase, hydrolase, and depolymerase, expressed by rare taxa. The expression of virulence factors and mechanisms of antimicrobial resistance suggested pathogenic genera were active, despite representing a minor component of the plastisphere community.

**Conclusion:**

Our study addresses a critical gap in understanding the functioning of the marine plastisphere, contributing new insights into the function and ecology of an emerging and important microbial niche. Our comprehensive multi-omics and comparative metaproteomics experimental design enhances biological interpretations to provide new perspectives on microorganisms of potential biotechnological significance beyond biodegradation and to improve the assessment of the risks associated with microorganisms colonizing marine plastic pollution.

Video Abstract

**Supplementary Information:**

The online version contains supplementary material available at 10.1186/s40168-024-01751-x.

## Background

Persistent plastic pollution represents a novel niche within the marine environment, providing an attractive surface for biofilm formation by marine microorganisms in an otherwise pelagic realm [1]. In recent years, the marine ‘plastisphere’ has become an intensely studied habitat with hundreds of studies reporting on the diversity and composition of the microbiome colonizing plastic surfaces [2]. The plastisphere is regarded as a complex interactome between the plastic, eukaryotes, bacteria, and archaea, with communities that can be taxonomically highly diverse [3, 4]. Many of the identified lineages display a proclivity for biofilm formation in marine systems, regardless of substrate type [5, 6]. However, some microorganisms may play an active role in plastic biodegradation [7]. Indeed, a recent meta-analysis revealed the cosmopolitan distribution of hydrocarbonoclastic *Oceanospirillales* and *Alteromonadales* on plastic pollution across discrete aquatic environments [8], suggesting that specific adaptations of these lineages enable the exploitation of the plastic niche. Other members of the plastisphere are potential pathogens displaying resistance to a range of common antibiotics [9] which may negatively impact ecosystem and human health [10, 11]. Yet, important questions regarding the extent of in situ biodegradation, the virulence of the identified pathogenic species, and the associated risks of the spread of antimicrobial resistance remain to be adequately addressed [9, 12–15]. Underpinning these knowledge gaps is the limited number of studies that have characterized the metabolic potential [16] and expressed functions of microorganisms associated with plastic pollution [7, 14, 15, 17].

To date, only two metaproteomic studies have been conducted on the marine plastisphere sampled directly from the environment [14, 15]. Both studies independently demonstrated the primary activity of photoautotrophic organisms, a diversity of active Eukaryotes, and indicated that although potential pathogens may be present, they were not metabolically active [14, 15]. Moreover, limited evidence exists of environmental microorganisms using plastic as a substrate for growth [13–15]. However, the impacts of environmental factors regulating marine biofilm growth, development, and metabolism, such as temperature, light intensity, and seasonality [18, 19], are poorly resolved for the functioning of the plastisphere. So far studies focusing on plastisphere metaproteomics have used plastic samples collected from warmer climates [14] or sampled during warmer seasons [15], which may explain the dominance by phototrophs and the regulation of phototroph activity on plastic debris [20]. By focusing future attention on both the taxonomic and functional diversity of the marine plastisphere, taking into consideration different locations, climates, and environmental regimes, significant new insights will be revealed into the functioning of microorganisms inhabiting this niche.

Through the quantification of expressed proteins, metaproteomics enables the elucidation of microbiome genotype–phenotype relationships to reveal active taxa and their functions [21]. When coupled with sample-specific genomics, theoretical proteomes, and data mining from public repositories, metaproteomics can significantly advance biological interpretations of even the most complex microbial communities [22]. A key aspect to accurately detecting microbial proteins is sufficient biomass of the active populations, in addition to the critical steps of protein separation, mass spectrometry, protein identification, and functional annotation through the generation of protein search databases [21, 23, 24]. For the plastisphere, an important facet to this is the optimization of the retrieval of the thin biofilm and subsequent cell lysis for optimal protein isolation [25, 26] and DNA co-extraction, which can be performed directly on plastic pieces or indirectly on physically dissociated cell pellets [14, 15]. To date, no comparison has been made as to the effectiveness of the different protein isolation, sample preparation, and protein annotation methodologies so far used to isolate and characterize expressed proteins from the plastisphere [14, 15]. Yet, these steps are vital to successfully connect plastisphere community structure to function using metaproteomics.

As plastisphere metaproteomics is still in its infancy, a critical assessment of the methodology is urgently required to optimize biological interpretations, and facilitate the adoption of this technique to expand our understanding of the functional repertoire of the plastisphere. In this study, a comprehensive experimental design was used to refine the key steps of (i) co-extraction (indirect versus direct), (ii) biofilm detachment and/or cell lysis (both mechanical and chemical), (iii) different proteomics workflows (gel-free versus gel-based), and (iv) the generation of protein search databases using, amplicon sequencing, metagenomics, and public repositories, to produce a combined optimal multi-omics and comparative metaproteomics workflow. We took the opportunity of this unique step-by-step recommendation to comprehensively study plastisphere function sampled from a temperate high latitude bathing beach, representing contrasting environmental conditions than those investigated previously using metaproteomics. Consequently, our study reveals the distinct activities of heterotrophic plastisphere lineages, as opposed to photoautotrophs, within a cold-adapted environment.

Collectively, in this study, we present (i) a critical assessment of DNA and protein co-extraction from plastic debris to facilitate future research, (ii) the dominant and active taxa present on plastic debris sampled in a high latitude cold climate, through examination of different proteomics and protein search database strategies, (iii) a novel perspective on plastisphere function driven by hydrocarbonoclastic lineages, and (iv) the use of a targeted approach to further resolve the specific biotechnologically and clinically relevant phenotypes of biodegradation and pathogenicity within the microbiome inhabiting marine plastic debris.

## Methods

### Sample collection

Marine plastic debris was collected from Gullane Beach, Scotland (56.0398° N, 2.8402° W), a designated bathing beach with ‘excellent’ water quality [27], during boreal autumn and spring (16/11/21 and 18/04/22). Debris was collected from the high tide mark along a 100-m transect following the westward expanse of the beach and placed into sterile sampling bags. Seawater temperature, dissolved oxygen, and salinity were measured using a handheld probe meter and a refractometer, respectively. Sampling conditions were consistent with the time of year and coastal location, whereby air temperature was 10.0 and 13.0 °C (boreal autumn or spring, respectively) and sea-surface temperature was 7.1 and 11.8 °C, respectively. Seawater salinity was ~ 36 PSU and dissolved oxygen measured 10.7 mg/L. Plastic debris was rinsed with artificial seawater and sorted based on their visual physical properties (e.g., morphology, color), dried briefly under laminar flow, and weighed, prior to storage at − 80 °C. A total dry weight of 58 g of plastic debris was collected, including 10.76 g of transparent films, 3.00 g of colored films, 8.96 g of colored fragments, 5.94 g of white fragments, 16.41 g of white polystyrene, and 13.01 g of colored foams.

### Indirect versus direct plastisphere co-extraction

**Fig. 1:**
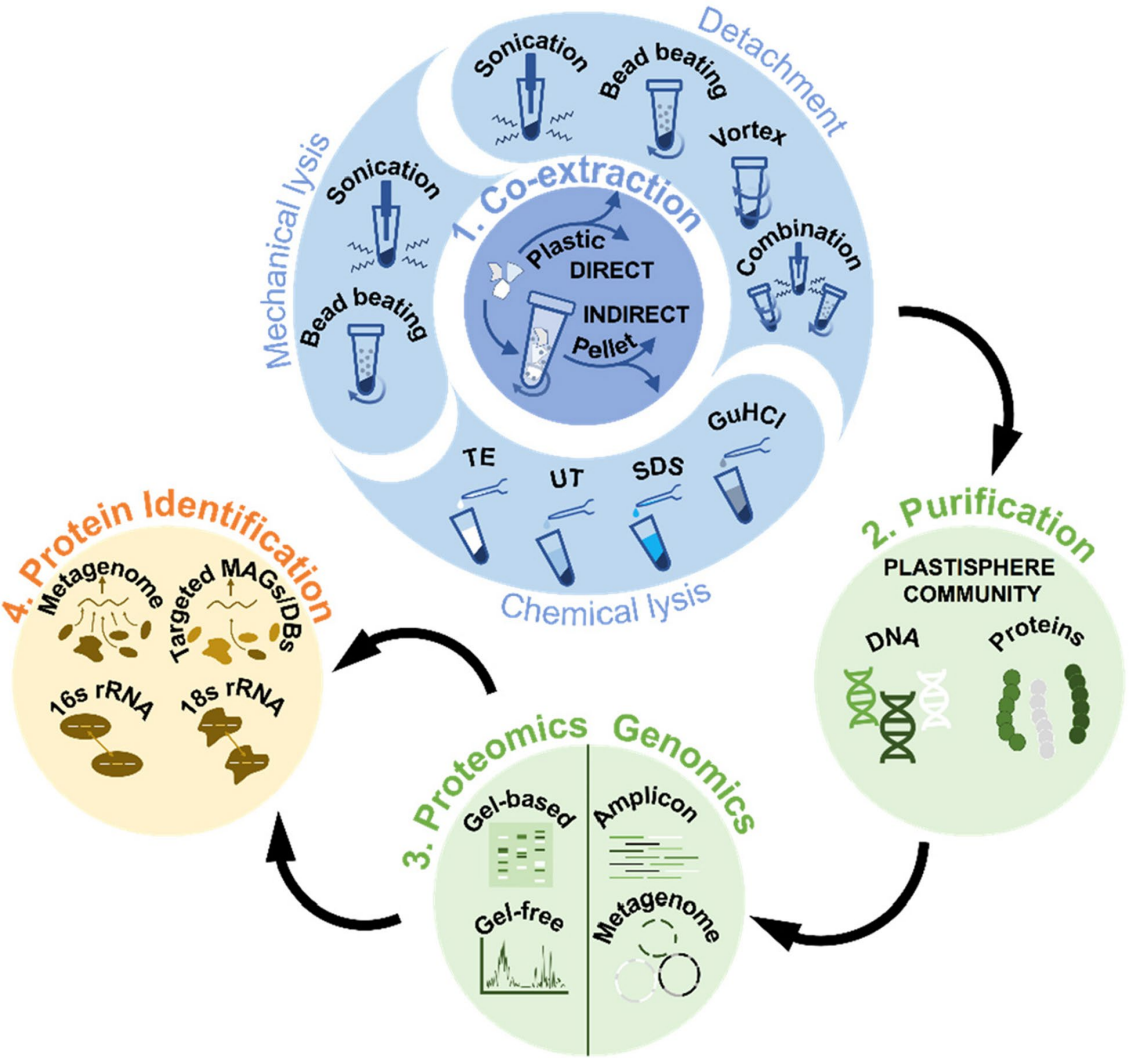
The critical steps assessed for the optimization of a plastisphere multi-omics and comparative metaproteomics workflow. TE = 10 mM Tris–HCl pH 7.5, 1 mM EDTA; SDS = 2% SDS, 50 mM Tris–HCl pH 7.5, 10 mM DTT; UT = 6 M urea, 2 M thiourea, 10 mM HEPES, 1 mM DTT; GuHCl = 6 M guanidine-HCl

Indirect and direct co-extraction of DNA and proteins was performed using the optimal detachment and cell lysis conditions (see below) on triplicate samples of transparent and colored plastic films (between 4.38 and 6.92 g). Briefly, three rounds of detachment via bead-beating (see below) were performed in either 15 ml complete artificial seawater [28] (ASW; indirect) or cell lysis buffer (direct) [2% SDS, 50 mM Tris–HCl pH 7.5, 10 mM DTT] (Fig. 1). Following the indirect workflow, the supernatant was recovered and centrifuged (12,000 × *g*, 15 min, 4 °C) and cells were resuspended in 2-ml cell lysis buffer. For direct cell lysis, the detachment supernatant was recovered prior to mechanical cell lysis. In both cases, plastisphere cells were mechanically lysed using sonication on ice (see below), cell debris was removed by centrifugation (8000 × *g*, 15 min, 4 °C), and the supernatant (retaining ~ 500 µl) was transferred to a 3 kDa ultra-filtration unit. Cell debris pellets were resuspended in the remaining ~ 500 µl of lysate, and sonication and centrifugation were repeated. The combined supernatants were concentrated by centrifugation (7000 rpm, 4 °C) until 250 µl remained, with 50 µl stored at − 80 °C for DNA co-extraction and proteins precipitated from the remaining sample for gel-free metaproteomics by incubation at − 80 °C overnight with ice-cold acetone (1:4 v/v).

### Mechanical indirect detachment of plastisphere cells or direct cell lysis

Four commonly used detachment approaches were tested on 2 g dry-weight mixed plastic debris (*n* = 3 per method) suspended in 20 ml complete ASW, namely (i) vortex, (ii) sonication, (iii) bead-beating, and (iv) combination of vortex, sonication, and bead-beating. Specifically including 3 min vortex on maximum speed [17], 2 min probe sonication [15] (1 s pulse, 1 s gap, 70% amplitude), 10 min bead-beating at maximum speed (1 g of 1 mm glass beads), and the combination of these. ASW was chosen for biofilm detachment to maintain the physiological integrity of cells. Control samples included triplicate 2 g dried plastic debris in ASW on ice. The success of cell detachment was measured via the increase in optical density (OD_600nm_) in 100 µl of supernatant, and the reduction in plastic-bound biomass using a crystal violet biofilm assay (OD_595nm_) [29], relative to the control. The viability of the detached cells (2 ml) was determined through incubation in marine broth (MB) for 24 h, monitoring optical density (OD_600nm_) at the start and end of the incubation as an indicator of growth. The optimal detachment method was then repeated three times to determine whether multiple rounds improved biofilm recovery.

Two mechanical approaches were tested to determine their effectiveness at cell lysis. After detachment, cells were pelleted by centrifugation (12,000 × *g*, 15 min, 4 °C) and resuspended in 2 ml urea-thiourea lysis buffer [6 M urea, 2 M thiourea, 10 mM HEPES, 1 mM DTT] [24] to facilitate protein solubilization. The suspension was subsequently aliquoted, with 10 min bead-beating (1 g 1 mm and 0.5 g ≤ 106 µm glass beads) performed at maximum speed on one aliquot, and repeated probe sonication (1 s gap, 1 s pulse, 40% amplitude, 2 × 1 min) performed on ice on the second. After mechanical lysis, cell debris was pelleted via centrifugation, concentrated, and acetone precipitated as described.

### Chemical cell lysis

Four cell lysis solutions were tested on 2-g dried mass mixed plastic debris (*n* = 3). Briefly, plastics were submerged in 15 ml ASW and subjected to three rounds of bead-beating for plastisphere detachment as above, and the supernatants were mixed by vortex for 30 s and subsampled into four, to produce one sample per cell lysis buffer, per replicate. Cells were pelleted via centrifugation (12,000 × *g*, 15 min, 4 °C) and resuspended in 2 ml of the following: (i) TE [10 mM Tris–HCl pH 7.5, 1 mM EDTA] [15], (ii) urea-thiourea [6 M urea, 2 M thiourea, 10 mM HEPES, 1 mM DTT] [24], (iii) SDS [2% SDS, 50 mM Tris–HCl pH 7.5, 10 mM DTT] [25, 30], and (iv) guanidine HCl [6 M] [14]. Cells were mechanically lysed using probe sonication and proteins precipitated as described.

### Protein recovery and quantification

Acetone-precipitated proteins were collected via centrifugation (16,000 × *g*, 15 min, 4 °C), washed twice with 100 µl ice-cold acetone to prevent cell lysis buffer contamination [25], and resuspended in 30 µl urea-ammonium bicarbonate buffer [8 M urea, 50-mM ammonium bicarbonate]. Insoluble material was removed by sonication (10 s; 1 s gap, 1 s pulse, 40% amplitude) and centrifugation (13,000 rpm, 15 min, 4 °C) [24], prior to sample dilution to reduce the urea concentration to 2 M. Total protein concentration was determined by Bradford Assay with bovine-gamma-globulin as a protein standard, and cell lysis and protein resuspension buffers were included as controls. Protein concentrations were qualitatively assessed by SDS-PAGE on 20 µg protein per sample, using a NuPage 4–12% Bis–Tris Gel (Invitrogen), prepared in accordance with the manufacturer’s instructions and visualized with Coomassie staining.

### Gel-free vs gel-based liquid chromatography-tandem mass spectrometry

Gel-free and gel-based proteomics were performed using protein isolates from the indirect and direct co-extraction samples employing the optimal detachment and cell lysis workflow. For gel-free proteomics, isolated proteins (20 µg) were reduced, alkylated, and precipitated with acetone prior to overnight trypsin digestion (1:25 enzyme/substrate ratio) at 37 °C, as previously described [24]. The trypsin digestions were terminated with 0.1% formic acid (final concentration). Samples were prepared for gel-based proteomics by SDS-PAGE as above, and 10 resolved bands were excised from one indirect and one direct co-extraction sample using a sterile scalpel. Gel fragments were washed twice with ammonium bicarbonate (25 mM; 10 min) followed by 50% acetonitrile (25 mM ammonium bicarbonate; 10 min, 300 rpm shaking, room temperature). Proteins were reduced in freshly prepared dithiothreitol (50 mM DTT; 25 mM ammonium bicarbonate) for 30 min at 60 °C and 300 rpm, followed by a repeat of the three washing steps. Reduced proteins were alkylated with Iodoacetamide (50 mM; 25 mM ammonium bicarbonate) for 30 min at 60 °C in the dark at 300 rpm. The three washing steps were repeated prior to overnight trypsin digestion at 37 °C and 300 rpm, with an enzyme/substrate ratio of 1:50.

Protein samples were analyzed on an ultra-high-performance liquid chromatography–high-resolution tandem mass spectroscopy (UHPLC-HRMS/MS) system, including a Eksigent NanoLC 400 and AB SCIEX TripleTOF 6600. Two micrograms of peptides were analyzed using acquisition parameters previously reported [31], and MS/MS spectra were acquired with the instrument operating in data-dependent acquisition (DDA), with micro-injection (75 min LC separation) modes.

### DNA isolation, sequencing, and analysis

To recover DNA from the cell lysate (see above), the protein was removed using protein precipitation solution (1:0.5 v/v; Promega; 5 min on ice) followed by centrifugation (16,000 × *g*, 15 min, 4 °C). Sodium acetate (3 M, pH 5.2; 1:10 v/v) and ≥ 95% ethanol (3:1 v/v) were added to the aspirated supernatant, mixed by vortex, and DNA precipitated at -20 °C overnight. DNA was recovered by centrifugation (16,000 × *g*, 15 min, 4 °C), and pellets were washed twice with fresh 70% ethanol. After centrifugation, residual ethanol was removed, and DNA resuspended in 20 µl Ultra-Pure water, with 10 min at 55 °C and 5 min on ice, to facilitate dissolution.

Microbial community composition was determined by 16S and 18S rRNA gene sequencing using primers targeting the V4–V5 regions of the 16S rRNA gene (515F-Y 5′-GTGYCAGCMGCCGCGGTAA-3′, 909R 5′-CCCCGYCAATTCMTTTRAGT-3′) [32–35] and the V8–V9 regions of the 18S rRNA gene (1422F 5′-ATAACAGGTCTGTGATGCCCT-3′, 1797R 5′-CCTTCYGCAGGTTCACCTAC-3′) [36, 37]. Samples were sequenced with negative and positive controls included on the Illumina MiSeq (2 × 300 nt paired-end) platform with V3 chemistry (StarSEQ® GmbH, Germany) and analyzed as Amplicon Sequence Variants (ASVs) using QIIME2 (v2022.2) [38] (see Supplementary Information for further details).

To ensure sufficient DNA for each treatment (indirect and direct), plastisphere genomic DNA was pooled for metagenomic sequencing on the Oxford Nanopore (ONT) MinION (Mk1C), following the manufacturer’s instructions (see Supplementary Information for further details). Quality-controlled reads were combined and taxonomically annotated using Kaiju [39], co-assembled into contiguous sequences using MetaFlye [40], binned into metagenome-assembled genomes (MAGs) using CONCOCT [41], then taxonomically and functionally annotated leveraging the KBase interactive metagenomics environment [42] (Department of Energy Systems Biology Knowledgebase, USA; see Supplementary Information for further details). Metabolic processes of interest, namely plastic biodegradation (PlasticDB) [43], virulence factors (VFDB) [44], and antimicrobial resistance (CARD) [45], were explored through alignment of the reads using Minimap2 [46] with default parameters.

### Protein search database creation, protein identification, and annotation

The generation of protein search databases utilized amplicon and metagenomic sequence data, and databases from public repositories. Briefly, 16S and 18S rRNA genus-level taxonomic assignments of ASVs, and genus-level taxonomic assignments of metagenomic reads combined with the annotated assembly, were used as input to generate three non-redundant protein search databases (16S-TaxDB (size = 7.3 Gb), 18S-TaxDB (19 Gb), and MG-DB (4.4 Gb), respectively) using the mPies database_creation workflow (v1.0) [47]. Briefly, mPies retrieves all available proteomes of identified taxa from the Uniprot database and removes redundances (100% sequence similarity) from the final protein search database [47]. To test a ‘second-search’ strategy [14, 24], a database was created from the high confidence genus-level taxonomic assignments of the 16S-TaxDB identified proteins (hereafter, 16S-TaxDB-2nd, size = 6.7 Gb), determined using the mPies annotate workflow (see below). The final protein search strategy encompassed the targeted identification of proteins using PlasticDB [43], VFDB [44], and CARD [45] as reference databases in independent searches and genome-resolved metaproteomics of the dominant *Pseudoalteromonas* sp. MAG recovered from the metagenome. The search databases were used within ProteinPilot (v5.0.3.1029, 9521aa4603a; Paragon Algorithm: 5.0.3.1029, 1029; AB SCIEX) via the OneOmics software package to identify protein groups with the parameters: sample types: identification, cysteine alkylation: iodoacetamide, digestion: trypsin, ID focus: biological modifications and amino acid substitutions, search effort: thorough ID, detected protein threshold [Unused ProtScore (Conf)] > : 0.05 (10.0%), false discovery rate analysis: at the protein level with a global threshold of 1%. Protein matches with only one peptide were manually curated to ensure ≥ 5 consecutive sequence-specific b- and y-type ions in series [24]. Proteins identified using 16S-TaxDB, 18S-TaxDB, MG-DB, and 16S-TaxDB-2nd were given a consensus taxonomic and functional annotation using mPies, based on the Lowest Common Ancestor (LCA) and DIAMOND BLAST respectively, as previously described [47]. To confirm the annotations of the targeted search databases, consensus functional and taxonomic annotations were obtained from the identified protein sequences using BLASTP against the NCBI nr database, with default parameters (last accessed May 18, 2023).

### Statistical analyses

Significant differences between treatments in recovered OD_600nm_, OD_595nm_, DNA and protein concentrations, and protein and peptide identification rates, were determined using a one-sided *T* test in R (v4.2.0). Significance tests on microbial community alpha diversity metrics were conducted in QIIME2 using pairwise Kruskal–Wallis tests as implemented in the q2-diversity plugin. Investigation of differentially abundant ASVs was conducted using QIIME2’s implementation of Analysis of Composition of Microbiomes (ANCOM) [48] on nonrarefied data using the composition plugin to add pseudocounts in replace of zeros. ASV by sample tables were exported from QIIME2 and converted to relative abundances, prior to Hellinger transformation and Bray–Curtis distance matrix generation for input into PERMANOVA using the vegan R package (v2.6–2) [49]. Identification of significantly up or down regulated proteins in the metaproteomic data was determined using a *T* test with examination of fold change in the MSstats package [50], after importing ProteinPilot.group files into Skyline and performing spectra integration, normalization, and relative quantification [51]. An adjusted *P* value of < 0.05 and fold change of 2 was considered significant.

## Results

### Direct co-extraction improves total DNA and protein yields, but not prokaryotic protein identification rates

A critical assessment of the key steps of a multi-omics and comparative metaproteomics workflow (Fig. 1) was undertaken to enhance biological interpretations of the functioning of the marine plastisphere. The effect of indirect (*n* = 3) and direct (*n* = 3) plastisphere co-extraction on DNA and protein yields, and the representation of the plastisphere community, was compared for the optimal biofilm detachment and cell lysis protocol. Although the results were variable, direct extraction resulted in mean total DNA concentrations ~ four-times greater than indirect extraction, at 6.2 ± 5.1 µg and 1.7 ± 1.1 µg, respectively (Table 1). Consistent with the DNA isolation results, direct extraction resulted in mean total protein concentrations that were ~ five times greater than indirect extraction, at 3350 ± 1991 µg and 674 ± 228 µg, respectively (Table 1). However, the opposite trend was observed in the protein identification results, whereby the numbers of proteins and distinct peptides identified using gel-free proteomics were generally higher in the indirect extraction samples, but these differences were not statistically significant (*T* test, *P* > 0.05; Table 1; Fig. S1). The mean coverages of identified peptide spectra, although relatively low, were significantly higher for the indirect extraction samples for three of the protein search databases (16S-TaxDB, 16S-TaxDB-2nd, MG-DB; *T* test, *P* < 0.05; Fig. S1).

**Table 1:** Yields from indirect and direct plastisphere co-extraction, including mean total DNA and protein (± standard deviation; S.D.), and mean protein and peptide identification rates from individual gel-free metaproteomes (*n* = 3 per treatment) across four search databases (16S-TaxDB, 18S-TaxDB, MG-DB, and 16S-TaxDB-2nd)

Variable (mean ± S.D.)	Indirect	Direct
Total DNA (µg)	1.7 ± 1.1	6.2 ± 5.1
Total protein (µg)	674 ± 228	3350 ± 1991
No. proteins (all DBs)	308 ± 88	175 ± 100
No. peptides (all DBs)	685 ± 327	356 ± 313

### Consistent taxonomy and function between indirect and direct extraction

**Fig. 2:**
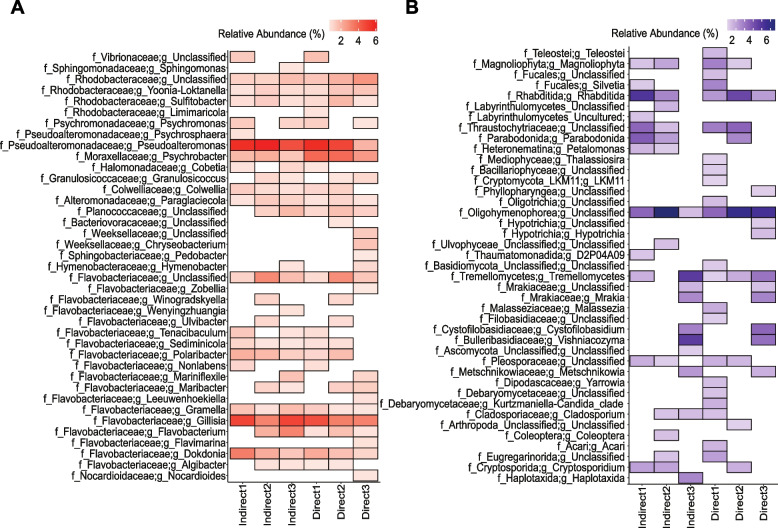
Plastisphere community composition between indirect and direct co-extraction for 16S rRNA (**A**) and 18S rRNA (**B**) genes. For visualization, amplicon sequence variants were collapsed into their respective genera, with only family and genus-level taxonomic assignments shown. Relative abundances were filtered to >0.1% of sequences and are Hellinger transformed for visual clarity

Despite the observed differences in DNA concentrations, sequencing of the 16S and 18S rRNA genes revealed no significant differences in the alpha diversity of the prokaryotic and eukaryotic plastisphere between indirect and direct extraction. This was evident for Faith’s Phylogenetic Diversity, Shannon’s diversity, and Pielou’s community evenness of both the 16S and 18S ASVs (Kruskal–Wallis, *P* > 0.05; *n* = 3 per treatment, per gene). Moreover, no significant differences were observed in the composition of 16S and 18S rRNA ASVs between the indirect and direct extraction samples (Fig. 2), for both compositional (ANCOM, *P* > 0.05; *n* = 3 per treatment, per gene) and relative abundances (PERMANOVA, *P* > 0.05; *n* = 3 per treatment, per gene). For both extraction types, the prokaryotic plastisphere was dominated by the phyla *Proteobacteria* and *Bacteroidota*, with ASVs taxonomically identified as *Pseudoalteromonas* sp., *Psychrobacter* sp., *Gillisia* sp., and *Dokdonia* sp., respectively, displaying prevalence across all samples (Fig. 2A). Although not statistically significant, eukaryotic ASVs displayed variability in their prevalence and relative abundances across replicates (Fig. 2B). Nevertheless, the dominant eukaryotic lineages belonged to the phyla *Basidiomycota*, *Ciliophora*, and *Nematozoa*, with ASVs identified as *Tremellomycetes* sp., unclassified *Oligohymenophorea*, and *Rhabditida Pellioditis marina* more consistent across samples (Fig. 2B).

To determine the impact of extraction type on plastisphere function, the relative abundances of proteins were explored using fold change and revealed few significant differences (Fig. S2). Specifically, only three proteins were significantly differentially abundant in the indirect extraction samples (identified using 16S-TaxDB). These included an unannotated protein, a *Pseudoalteromonas* sp. 50S ribosomal protein, and a *Flavobacterium* sp. *BFFFF2* Bacterial Ig-like domain (Big_12 domain-containing protein). Conversely, only one protein annotated as *Psychrobacter* sp*.* Elongation factor G was significantly differentially abundant in direct extraction. No statistically significant differences in the relative abundance of proteins identified as eukaryotic (identified using 18S-TaxDB) were observed between the extraction types.

### Mechanical detachment and cell lysis approaches impact plastisphere recovery and protein yields

To standardize biofilm detachment across different plastic properties and polymer compositions, four mechanical approaches were tested on 2 g mixed plastic debris. All approaches resulted in significant detachment relative to the control, with the highest optical densities of recovered cells observed in the vortex, bead-beating, and combination treatments (Fig. S3A). Within these, bead-beating was the most reproducible and displayed the greatest difference relative to the control (*T* test, *P* = 0.0003; Fig. S3A) and the greatest decrease in plastic-bound biofilm (reduction in the range 0.163–0.171 OD_595nm_; Fig. S3B). Three rounds of bead-beating further improved cell recovery relative to the control (data not shown). For all methods, plastisphere cells detached in ASW remained viable, demonstrating growth after 24 h in nutrient-rich media (Fig. S4A).

Two common mechanical cell lysis approaches, namely bead-beating and sonication, were tested on cells detached from the plastisphere. Significantly greater mean total protein yields were observed following sonication compared to bead-beating, at 378 µg and 93 µg, respectively (Fig. S3C; *T* test, *P* < 0.05). Subsequently, four chemical cell lysis buffers were tested in combination with sonication revealing differences in cell lysis efficiency and protein recovery, such that TE < urea-thiourea < guanidine HCl < SDS (Fig. S3D). The TE-based buffer was the least efficient, with normalized median yields of 55 µg g^−1^ plastic debris, compared to median yields of 192 µg g^−1^ plastic using the most efficient SDS-based lysis buffer (Fig. S3D). Cells lysed by sonication in TE buffer remained viable after 24 h growth in nutrient-rich media, while those lysed in the SDS-, urea-thiourea-, and guanidine HCl-based buffers were no longer viable (Fig. S4B).

### Complementary gel-free and gel-based metaproteomics

**Fig. 3:**
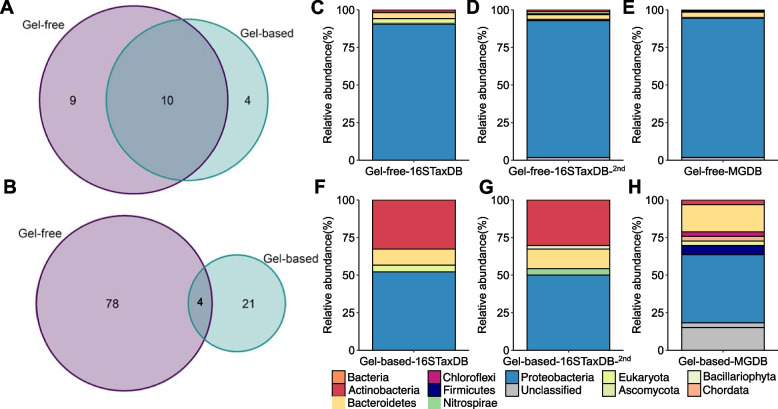
Comparison of annotated proteins from the gel-free and gel-based metaproteomes. Numbers of shared and unique genera-level taxonomic annotations (**A**) and protein functions (**B**), phylum-level taxonomic annotations of identified proteins in the gel-free (**C**, **D**, **E**), and gel-based (**F**, **G**, **H**) metaproteomes using different search databases

To evaluate gel-free and gel-based protein fractionation, protein identification rates and their taxonomic and functional annotations were qualitatively compared between six combined gel-free and 20 combined gel-based metaproteomes (Fig. 3). Overall, protein identification rates were higher in the combined gel-free metaproteome, with 967 distinct proteins and 1506 distinct peptides, compared to 382 proteins and 391 distinct peptides in the gel-based approach (16S-TaxDB; including peptides ≥ 1). Following taxonomic annotation of the identified proteins, 10 unique genera were found to be shared between the gel-free and gel-based metaproteomes, including those attributed to the dominant lineages, *Pseudoalteromonas*, *Pseudomonas*, and *Psychrobacter*, and the less prevalent, *Marinobacter*, *Sphingomonas*, *Streptomyces*, and *Vibrio*. While 9 genera were specific to the gel-free, only 4 were unique to the gel-based approach (Fig. 3A), namely actinobacterial *Kitasatospora* and *Nocardioides*, and gammaproteobacterial *Lysobacter* and *Shewanella*. Interestingly, the functional annotations reflected divergence between the gel-free and gel-based approaches (Fig. S5A-B), with only 4 shared unique protein functions between them (Fig. 3B). The shared proteins were associated with bacterial translation, ribosomal structure, and biogenesis (3 unique functions) and a periplasmic substrate-binding protein involved in inorganic ion transport and metabolism. A total of 78 unique protein functions were identified specifically using the gel-free approach, with the majority of these spanning the functional categories of translation, ribosomal structure and biogenesis (29 unique functions), energy production and conversion (15 unique functions), and posttranslational modification, protein turnover, and chaperones (7 unique functions) (Fig. S5A). Conversely, 21 unique protein functions were specific to the gel-based proteomes, with the most common functional categories encompassing amino acid transport and metabolism (3 unique functions), signal transduction mechanisms (3 unique functions), carbohydrate transport and metabolism (2 unique functions), cell wall biogenesis (2 unique functions), and transcription (2 unique functions) (Fig. S5B).

### The role of protein search database in determining plastisphere structure and function

**Fig. 4:**
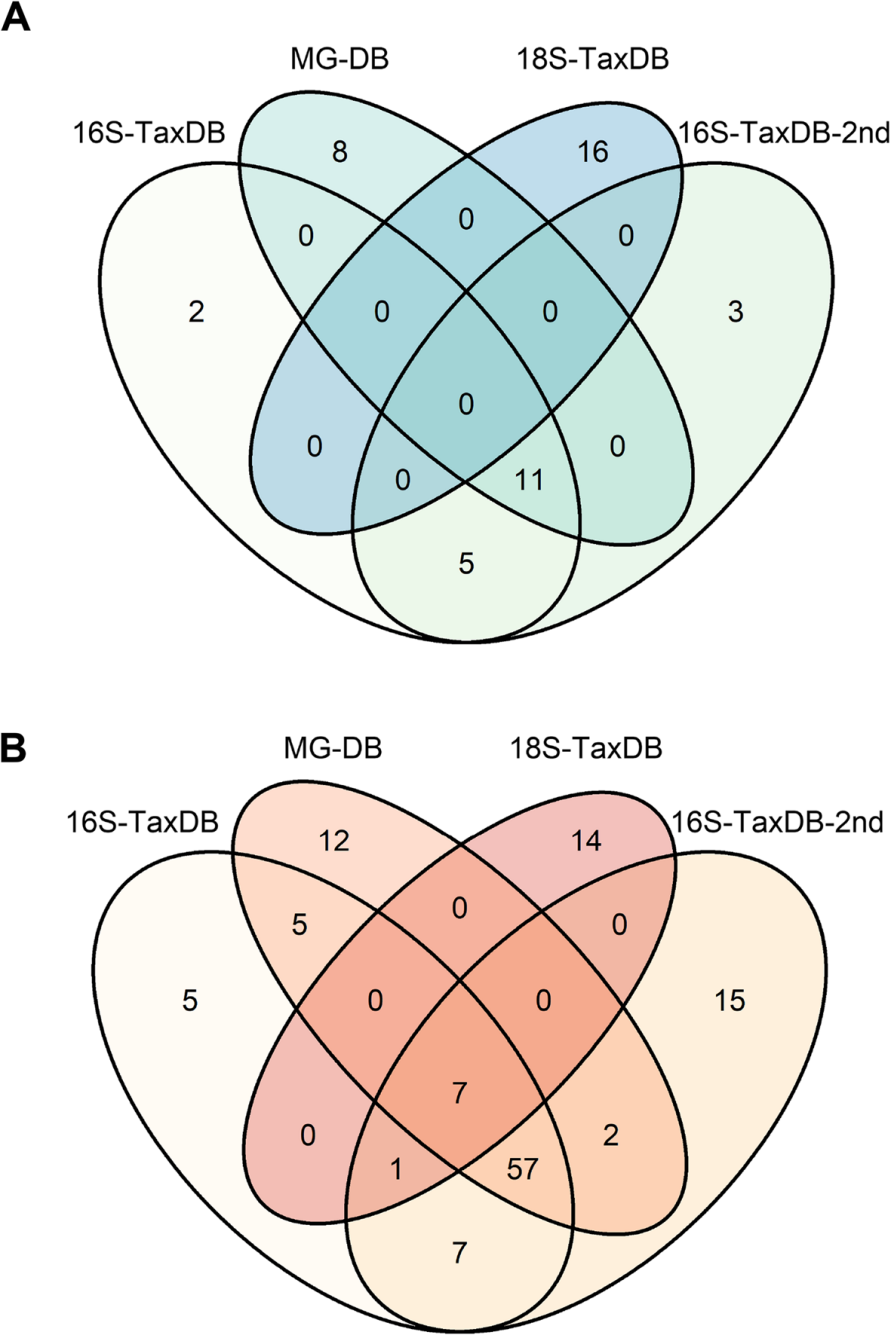
Comparison of annotated proteins from different search strategies reveals differences in the identification of unique genera (**A**) and unique protein functions (**B**)

Comparison of the high confidence protein annotations revealed similarities and differences in the diversity of the active taxa identified using the 16S-TaxDB, the second search database, 16S-TaxDB-2nd, and MG-DB (Fig. 3C–H; Fig. 4A). 16S-TaxDB recovered proteins from 5 phyla (4 Bacteria and 1 Eukaryota; Fig. 3C, F) and 18 genera (Fig. 4A), while 16S-TaxDB-2nd detected 6 phyla (4 Bacteria and 2 Eukaryota; Fig. 3D, G) across 19 genera (Fig. 4A). MG-DB identified the greatest diversity of organisms spanning 7 phyla (3 Bacteria, 3 Eukaryota, and 1 viral; Fig. 3E, H) and 19 genera (Fig. 4A). In total, 11 genera were shared between 16S-TaxDB, MG-DB, and 16S-TaxDB-2nd (Fig. 4A), while 8 genera were unique to MG-DB, including *Bradyrhizobium*, *Cobetia*, *Gillisia*, *Granulosicoccus*, *Polaribacter*, *Phyllobacterium*, *Planococcus*, and the virus, *Prymnesiovirus*. In contrast, only 2 and 3 genera were specific to 16S-TaxDB and 16S-TaxDB-2nd, respectively (Fig. 4A). These included *Devosia* and *Marinobacter* for 16S-TaxDB and *Cylindrotheca*, *Halomonas*, and *Moraxella* for 16S-TaxDB-2nd. Additional active eukaryotic organisms were highlighted through protein identification using 18S-TaxDB; however, the taxonomic identities were largely unresolved by LCA, with only a few proteins of the phyla *Bacillariophyta* and *Oomycota* annotated. Taxonomic annotations from the DIAMOND BLAST results revealed 5 additional phyla spanning the *Arthropoda*, *Ascomycota*, *Chordata*, *Nematoda*, and *Streptophyta*. Although these classifications should be interpreted with caution due to the limitations of short peptide sequences, these contained 16 genera (Fig. 4A). Within the functional annotations, the numbers of total functional categories were greatest for proteins identified using the 16S-TaxDB-2nd, and MG-DB, followed by 16S-TaxDB, and 18S-TaxDB, at 18, 18, 17, and 6 unique categories, respectively. At the protein level, eukaryotic functional annotations were limited, but 7 unique protein functions were shared between all four search strategies, including ATP synthase subunits, 50S ribosomal proteins, elongation factors, and chaperonin proteins. 16S-TaxDB, MG-DB, and 16S-TaxDB-2nd shared 57 of the annotated unique protein functions. Across these individual search databases, 5, 12, and 15, unique protein functions were found to be specific to 16S-TaxDB, MG-DB, and 16S-TaxDB-2nd, respectively (Fig. 4B).

### Multi-omic insights into a heterotrophic plastisphere

**Fig. 5:**
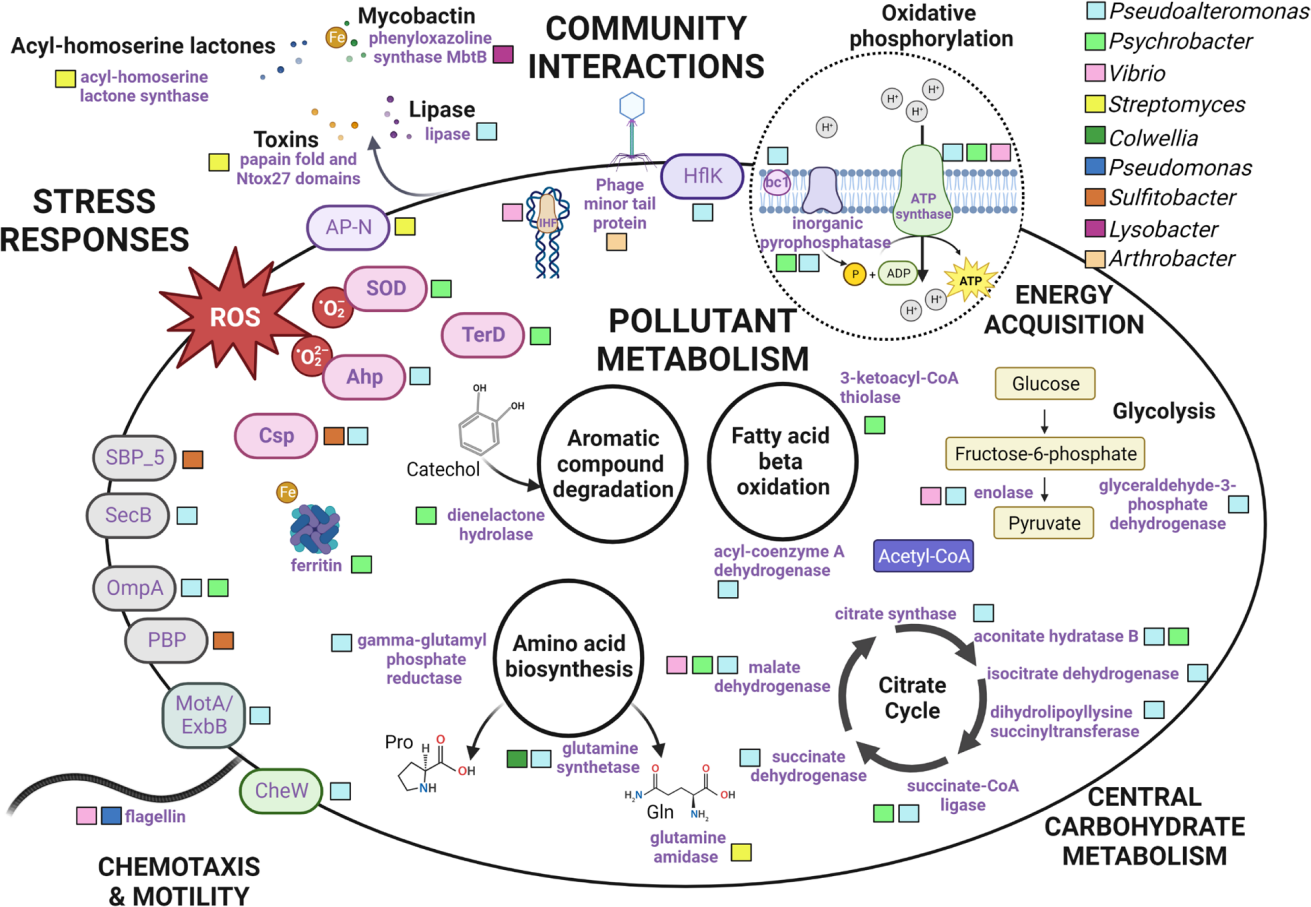
Metabolic overview of the key pathways identified within a cold-adapted plastisphere dominated by heterotrophic microorganisms. The schematic representation of the expressed pathways and the taxonomic assignment of proteins is based on the MG-DB annotated gel-free metaproteomes. Full details are provided in Table S1

Annotation of the metabolic potential within the representative metagenome (*n* = 1), combined with the expressed proteins identified using the most comprehensive gel-free metaproteome annotations (*n* = 6; 16S-TaxDB, MG-DB, and 16S-TaxDB-2nd; Fig. 4), provided insights into the functioning of the heterotrophic plastisphere. The predicted metabolism, guided by the metagenome, revealed genes encoding energy acquisition, carbon metabolism, and amino acid metabolism, which were complemented by a range of membrane transporters (Fig. S6A-D). The metaproteomes revealed the specific expression of proteins involved in oxidative phosphorylation, the citrate cycle, glutamine and proline biosynthesis, and carbohydrate metabolism, providing confirmation of the importance of these pathways (Fig. 5; Table S1). Interestingly, proteins associated with stress responses were a prominent feature of the metaproteomes, including the specific expression of TerD (*Psychrobacter*), superoxide dismutase (*Psychrobacter*, *Planococcus*), alkyl hydroperoxide reductase (*Pseudoalteromonas*), ferritin (*Psychrobacter*), and cold-shock and heat-shock proteins in several lineages (Fig. 5). Both the metagenome and metaproteomes included mechanisms associated with biofilm formation, such as motility, chemotaxis, and adhesion, with several expressed proteins also associated with virulence factors, such as *Pseudoalteromonas* lipase, *Lysobacter* mycobactin siderophore (Phenyloxazoline synthase MbtB), elongation factors, *Pseudomonas* type II secretion, and *Streptomyces* toxins (papain fold toxin domain, Ntox27 domain-containing protein; Fig. 5; Table S1). Furthermore, proteins indicative of inter-community interactions were also expressed, including those mediating protein–protein interactions in *Flavobacterium* (Big_12 domain), quorum sensing in *Streptomyces* (acyl-homoserine lactone synthase), and others suggestive of viral infection in *Arthrobacter*, *Vibrio*, and *Pseudoalteromonas*, such as a phage-related minor tail protein, an integration host factor, and HflK (Fig. 5; Table S1). Notably, evidence of genes and expressed proteins from pathways relevant to marine pollutants, such as aromatic compound degradation in *Psychrobacter* (e.g., homoprotocatechuate and chlorocatechol degradation) and fatty acid beta-oxidation in *Psychrobacter* and *Pseudoalteromonas* (alcohol dehydrogenase, 3-ketoacyl-CoA thiolase, acyl-coenzyme A dehydrogenase), were provided by both approaches (Fig. 5; Table S1). Unsurprisingly, genes and proteins associated with information systems were also highly prevalent, such as those associated with ribosomes, chaperonins, transcription, and translation (Table S1).

The taxonomic annotations of the multi-omic datasets revealed the dominance of Proteobacteria, in particular, *Pseudoalteromonas* (Fig. 2A; Fig. 3; Table S2; Fig. 5). Metagenomic binning resulted in one partial MAG identified as *Pseudoalteromonas* sp. (77% completeness, < 10% contamination, including 13 total rRNAs and 97 tRNAs), and the predicted proteome was used as a reference database to further resolve this microorganism’s activity. This resulted in the identification of 98 expressed proteins and 431 high-confidence peptides for relative quantification (Table S3). The most abundant proteins expressed by *Pseudoalteromonas* sp. were involved in general metabolism and energy acquisition, including a peptidylprolyl isomerase (FkpA) involved in protein folding, large and small subunit ribosomal proteins, translation factors, RNA polymerases, and energy acquisition via oxidative phosphorylation, which were present at between 1 and 2.5% mean relative abundances. Several proteins involved in carbohydrate metabolism were also expressed, with proteins of the citrate cycle representing 4.5% of total mean relative abundance and two proteins involved in glycine metabolism also present. Besides these metabolic processes, proteins involved in membrane transport, such as the OmpF porin, TonB-dependent receptor, and the ExbB biopolymer transport protein, a carboxypeptidase regulatory-like domain, and a cation/acetate symporter, collectively represented a total of 3.6% mean relative abundance. Elongation factor Tu, which is integral to prokaryotic translation, and plays potential roles in cell-surface adhesion and virulence [52], and chemotaxis proteins (cheW, and, methyl-accepting chemotaxis protein) were also detected at mean relative abundances of 2.3 and 0.3%, respectively (Table S3). Interestingly, proteins associated with DNA damage/repair, including an excision nuclease, and oxidative stress response, such as thioredoxin and cold-shock protein, were also expressed (Table S1; Table S3; Fig. 5).

### A targeted approach to detect polymer degradation and pathogenicity

Targeted protein searches with public databases confirmed the presence and expression of two key plastisphere phenotypes indicated within the multi-omic data: polymer degradation and pathogenicity (virulence and antimicrobial resistance). Alignment of the metagenomic reads to PlasticDB revealed the presence of 5 polymer degradation genes, which included a laccase, depolymerases, an esterase, and dehydrogenase from a range of taxa present within the plastisphere (Table 2). Of these, 36% were annotated as the cold-adapted *Psychrobacter* sp*.* laccase, which mediates polyethylene degradation [53]. Annotation and relative quantification of the metaproteomes using PlasticDB identified 3 expressed proteins associated with polymer degradation from the *Actinomycetia* and *Betaproteobacteria* (Table 2). These included nylon-degrading polyamidase (*Nocardia* sp.) and hydrolase (*Paenarthrobacter* sp.), which represented ~ 60% of the PlasticDB quantified proteins, and a poly lactic acid depolymerase (*Paucimonas lemoignei*) (Table 2). These taxa were identified within the metagenome at low relative abundances (Table S2).

**Table 2:** Multi-omic investigation of polymer degradation within the plastisphere. Annotations and relative abundances are based on the number of aligned metagenomic reads, or relative quantification of proteins, as a proportion of the total identified using PlasticDB as a reference

Metagenome					
**PlasticDB enzyme**	**Substrate**	**PlasticDB species**	**PlasticDB phyla**	**Average ID (%)**	**Rel. ab. (%)**
Laccase	PE	*Psychrobacter* sp*.*	Proteobacteria	80.3	36.4
Depolymerase	PLA	*Marinobacter* sp.	Proteobacteria	79.8	18.2
Dehydrogenase	PHA, P3HV/PHBV	*Paracoccus denitrificans*	Proteobacteria	80.8	18.2
Esterase	PLA	Uncultured bacterium	Uncultured bacterium	83.3	18.2
Depolymerase	PLA	*Pseudomonas putida*	Proteobacteria	78.7	9.1
**Metaproteome**					
**PlasticDB Enzyme**	**Substrate**	**PlasticDB taxonomy**	**Protein**	**Consensus taxonomy**	**Rel. ab. (%)**
Depolymerase	PLA	*Paucimonas lemoignei*	Poly(3-hydroxyalkanoate) depolymerase C	*Paucimonas lemoignei*	12.4
Hydrolase	Nylon	*Paenarthrobacter ureafaciens*	6-aminohexanoate-dimer hydrolase	*Paenarthrobacter* sp.	28.3
Polyamidase	Nylon	*Nocardia farcinica*	6-aminohexanoate-cyclic-dimer hydrolase	*Nocardia* sp.	30.9

*PLA* Poly lactic acid, *PE* Polyethylene, *PHA* Polyhydroxyalkanoate, *PHB* Polyhydroxybutyrate, *P3HV/PHBV* Poly(3-hydroxybutyrate-co-3-hydroxyvalerate)

**Fig. 6:**
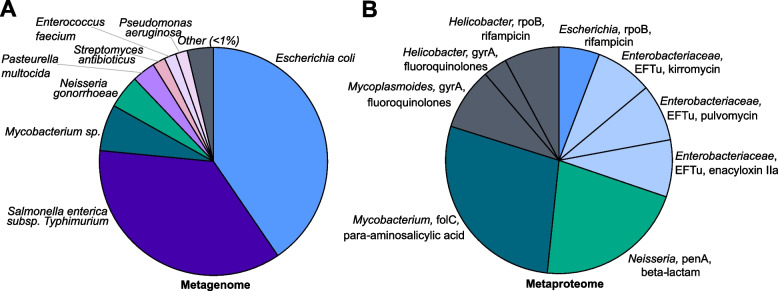
Identification of pathogens encoding mechanisms of antimicrobial resistance within the cold-adapted plastisphere. Proportions of different taxa within the metagenome reads aligned to CARD (**A**) and proportions of quantified expressed proteins and their resistance profiles (**B**)

Pathogenicity was explored through the targeted databases, VFDB and CARD. Alignment of metagenomic reads to VFDB resulted in 388.0 Kbp of sequence across 649 alignments. The most abundant virulence factors within the metagenome were associated with cell adherence and motility mechanisms, in *Francisella tularensis*, *Pseudomonas aeruginosa*, and *Mycoplasma hyopneumoniae* (Fig. S7). Besides the virulence factors identified using the MG-DB and 16S-TaxDB, protein identification using VFDB confirmed the expression of a *Francisella* sp. TufA protein and a B-type flagellin of *Pseudomonas aeruginosa*. Furthermore, antimicrobial resistance genes were identified through the alignment of the metagenomic reads to CARD, resulting in the identification of 123 genes across 7723 alignments. Taxonomically, these genes were associated with an array of pathogenic organisms present within the metagenome (Table S2), including *Escherichia coli*, *Salmonella enterica* serovar Typhimurium, and *Mycobacterium* sp. (Fig. 6A). Of the 123 CARD genes identified, 6 were designated as clinically relevant, including identification of *Mycobacterium smegmatis* tetracycline resistance (*tetV*), *E. coli* fluoroquinolone resistance (*oqxB*), and *Enterobacter aerogenes* erythromycin resistance (*qepA*). The use of the CARD protein variant model as a protein search database identified 11 expressed proteins for relative quantification, including those conferring resistance to rifampicin, beta-lactam, and fluoroquinolones in the bacterial genera *Escherichia*, *Helicobacter*, *Neisseria*, and *Mycoplasmoides* (Fig. 6B). In addition to resistance to kirromycin, pulvomycin, and enacyloxin IIa, within the Enterobacteriaceae (Fig. 6B).

## Discussion

### Optimal plastisphere co-extraction, biofilm detachment, and cell lysis

As the plastisphere is comprised of a diversity of Bacteria, Archaea, and Eukaryotes [4], and metaproteomics cannot include a target amplification step, accurate representation of plastisphere structure and function requires complete co-extraction of all biofilm cells. This is especially true for thin microbial biofilms, where biofilm growth and metabolism may be limited by light, temperature, nutrients, and other environmental factors [20, 54, 55], resulting in reduced biofilm thickness and active biomass [18]. Previously, plastisphere protein isolation has been accomplished either directly on plastic pieces [15], indirectly on detached cell pellets [14], or subsequently on cultured isolates [7, 17]. Plastisphere recovery may be facilitated by performing co-extraction with mechanical and chemical lysis directly on plastic pieces [15]. While indirect co-extraction enables the recovery of viable plastisphere cells for cultivation, as well as molecular analyses [7]. Our results indicated that indirect and direct co-extraction could be used interchangeably, with no apparent effect on the representation of plastisphere community structure or function. However, higher peptide spectra coverages and protein identification rates were observed following indirect extraction, leading to the hypothesis that direct co-extraction may increase nonprotein interference with the colorimetric Bradford Assay [56, 57] used for protein quantification (see Supplementary Information). Indeed, we confirmed protein yields via SDS-PAGE and identified an approximately fivefold overestimation of protein concentration based on the Bradford Assay. We highly recommend the use of SDS-PAGE to confirm protein yields from complex plastisphere samples in the future.

Detachment and extraction of the plastisphere have been achieved in previous studies using a range of mechanical and chemical means, such as sonication [7, 15], vortex [17], scraping of the biofilm [14], and SDS treatment [29]. Herein, bead-beating, which is widely used for sample homogenization and lysis [58], was determined to be a reproducible and efficient method of detachment, while cell lysis using sonication in an SDS-based buffer resulted in enhanced protein yields. This is in line with previous studies demonstrating the ability of sonication to effectively lyse diverse cell morphologies and cell wall compositions, such as gram-positive bacteria [25, 59]. Improved protein yields following the application of an SDS-based buffer, which is a strong ionic detergent that facilitates protein solubilization and inactivates some proteases, mirrors previous methodological comparisons of protein isolation from the human gut microbiome [25], marine sediments [60], and planktonic samples [23, 26]. A 1–2% SDS solution is typically used for protein isolation [25, 60, 61], though it must be noted that SDS can interfere with enzymatic digestion and mass spectrometry [62], and therefore, it should be diluted or removed during sample preparation. The optimized approach enabled the characterization of a diversity of active taxa, including gram-negative *Proteobacteria*, gram-positive *Actinobacteria*, in addition to the Eukaryotic *Bacillariophyta* and *Ascomycota*, suggesting differences in cell wall and/or membrane composition and attachment strategy, did not impair biofilm recovery using our robust lysis method.

### Complementary gel-free and gel-based metaproteomics

Metaproteomic studies typically employ gel-free or gel-based protein fractionation to reduce sample complexity prior to mass spectrometry [21, 63–65], but this may have downstream ramifications on the observed community structure and function [24]. In the present study, gel-free protein fractionation resulted in higher protein identification and annotation rates, in addition to revealing a greater diversity of active plastisphere taxa and protein functions, compared to gel-based proteomics. While both highlighted unique taxa and proteins, together they captured proteins from the most abundant active genera within the dominant *Proteobacteria*, such as *Pseudoalteromonas*, *Pseudomonas*, *Psychrobacter*, and *Vibrio*. Furthermore, each approach captured the expression of important metabolic functions, although the number of unique protein functions was greatly reduced in the gel-based metaproteomes. Previously, Oberbeckmann and colleagues [15] utilized a gel-based approach, analyzing 20 protein bands per plastisphere sample with > 60% of proteins derived from Eukaryotic lineages, primarily *Bacillariophyta* and *Arthropoda*. In contrast, Delacuvellerie et al. [14] employed a gel-free approach and primarily identified proteins derived from the phyla *Cyanobacteria* and *Proteobacteria*, and to a lesser extent *Bacillariophyta* and *Arthropoda*. Although limited in scope due to the scarcity of studies, these differences may represent methodological artifacts based on different protein fractionation strategies, whereby a gel-based approach may allow access to less abundant proteins from more specialized taxa, compared to gel-free proteomics which typically captures the most abundant proteins [21, 65]. Thus, consistent with previous research [21, 24], our findings suggest both approaches are complementary and will facilitate the identification of different proteins and active taxa in the marine plastisphere when applied more broadly across a range of plastic types and locations.

### A metagenome-derived protein search database improves protein identification

The optimization of a protein search database for protein identification and annotation was facilitated by the publicly available tool mPies [47] and highlighted the need to obtain sufficient coverage of the microbial community for detailed functional inferences. Previously, the use of a comparable metagenome to generate a protein search database has been shown to increase protein identification rates [24, 47, 61]. Here, the MG-DB resulted in improved spectra coverages, protein identification, and annotation rates compared to the 16S- and 18S-TaxDBs, revealing a diversity of active prokaryotic, eukaryotic, and even viral, members of the plastisphere. An additional effective strategy to improve protein identification rates is the application of a second-round search, in which only taxa identified in the first search are retained and used as a reference [14, 24]. This was marginally effective in the present study, resulting in heterogenous improvements in identified peptide spectra coverages and the numbers of identified and annotated proteins. Previously, Oberbeckmann and colleagues [15] reported difficulties in protein identification due to the presence of poorly characterized Eukaryotes within the marine plastisphere. We also observed that the annotation of Eukaryotic proteins was largely inconclusive using the robust LCA approach, and theoretical proteomes were scarce for many of the patchy eukaryotes identified within our 18S rRNA gene sequencing data; consequently, few of the proteins identified using 18S-TaxDB were annotated. As protein identification is dependent on the availability of the predicted proteome of the organisms within the sample, annotation may be improved by increasing the depth of metagenomic sequencing to capture more of the rare taxa, and those with large genome sizes, to include their encoded functions during database creation. However, increasing the protein search database size can reduce the sensitivity of peptide spectrum matches [66], and is therefore not guaranteed to improve protein identification. Since many marine plastisphere lineages have only been studied using low-resolution taxonomic markers [2], improved genomic representation through future studies may facilitate protein identification using novel bioinformatic tools [66]. Our results demonstrating a range of active lineages using different protein search strategies indicate a concerted effort should be made to better characterize the metabolic potential, not just the taxonomy, of plastisphere lineages in the future.

### Functioning of a heterotrophic plastisphere

The plastisphere has been described as a self-sufficient ecosystem [4]; a notion supported by the confirmed activity of primary producers, heterotrophs, and eukaryotes in previous studies [14, 15, 67]. Thus, understanding the overall functioning of this community and the specific lineages within it is important to ultimately reveal the ecology of this niche. Heterotrophic lineages from the *Gamma*- and *Alphaproteobacteria*, and to a lesser extent the *Flavobacteria*, were highly prevalent within our plastisphere samples, in contrast to eukaryotic lineages which displayed substantial variability between replicates, and this was reflected in the annotated plastisphere genes and proteins using multi-omics. Metabolic activity primarily driven by heterotrophic lineages contrasts with previous metaproteomic plastisphere studies in which photoautotrophic *Bacillariophyta* and *Cyanobacteria* dominated [14, 15]. We did not identify the presence or activity of cyanobacteria and detected only limited evidence of active *Bacillariophyta*. Correspondingly, energy acquisition through photosynthesis was not a prominent feature of our plastisphere metaproteomes; rather, the citrate cycle coupled with oxidative phosphorylation was the major metabolic pathway expressed to obtain energy. This heterotrophic, as opposed to photoautotrophic metabolism, may be selected for by the environmental conditions experienced during our boreal spring sampling [20], as a factor of spatial and temporal scales [18, 20, 54, 55, 68], or due to the chemical composition of the plastisphere itself [69]. Supporting this, evidence of adaptation to the plastisphere and chemical microenvironment was provided by several lineages, through the presence of cold-shock protein domains (*Rhodobacteraceae*, *Pseudoalteromonas*, *Phyllobacterium*), antioxidants which may mitigate the activity of reactive oxygen species generated through exposure to pollutants (*Psychrobacter*, *Planococcus*, *Pseudoalteromonas*), and proteins meditating pathways of xenobiotic degradation (*Psychrobacter*, *Pseudoalteromonas*).

Genome-resolved metaproteomics was used to identify the expressed proteins of the dominant lineage *Pseudoalteromonas* sp., within the plastisphere for the first time. Marine *Pseudoalteromonas* are well-known for their ecological and biotechnological significance, whereby they play important roles in biogeochemical nutrient cycling through the degradation and remineralization of marine polysaccharides and represent a group of widespread biofouling and antifouling species [6, 70, 71]. For these reasons, *Pseudoalteromonas* sp. may play a role in polymer degradation [1] or in symbiotic association with Eukaryotes such as diatoms [72]. We did not identify any *Pseudoalteromonas* sp. proteins involved in polymer degradation, but those encoding fatty acid beta-oxidation were expressed, and our results further revealed the expression of proteins involved in carbohydrate, amino acid, and nucleotide metabolism, in addition to membrane transport and chemotaxis. These functions may well reflect metabolic interactions between *Pseudoalteromonas* sp. and other plastisphere taxa [73]. We anticipate that future metaproteomic studies will highlight the specific functional roles of this group, and other key lineages within the marine plastisphere, shedding light on their metabolic and ecological interactions.

### Identification of polymer degradation and pathogenicity phenotypes

It has been argued that the marine plastisphere represents a generalist biofilm comprised of lineages that favor surface attachment over pelagic lifestyles [74], with limited evidence of the expression of functions related to the specific use of marine plastic as a substrate for growth [13–15]. However, members of the plastisphere involved in polymer degradation may represent rare taxa that have been previously overlooked in taxonomic investigations [75, 76], and underrepresented in functional studies. Indeed, metaproteomics of the marine plastisphere has so far reported the dominance of proteins involved in photosynthesis as opposed to polymer degradation [14, 15]. Herein, the dominant and active taxa identified using our optimized multi-omics workflow included the hydrocarbon-degrading lineages *Pseudoalteromonas*, *Pseudomonas*, *Flavobacterium*, and *Psychrobacter*, with hydrocarbonoclastic bacteria previously shown to be present at elevated abundances due to the history of oil exploration and production in the Northeast Atlantic proximal to Scotland [77]. Moreover, the presence of genes and the expression of proteins involved in aromatic compound degradation and fatty acid beta-oxidation hinted at the potential for polymer degradation, and a PE-degrading *Psychrobacter* sp*.* laccase was identified within our metagenome using PlasticDB. Further, the use of PlasticDB as a targeted protein search strategy provided evidence for the expression of a polyamidase, hydrolase, and depolymerase involved in polymer biodegradation. The proteins were taxonomically attributed to lineages representing only a small proportion of the present and active community members detected herein, supporting the hypothesis that these metabolisms are rare within the plastisphere but also likely under-reported [75, 76]. Although it is necessary to validate this targeted approach and confirm enzyme activity, these findings reflect the urgent need to determine the expression of important microbial functions, such as biodegradation, on field-collected plastisphere samples, to further refine our understanding of the fate of plastic in the environment.

Previous research has identified marine plastics as a potential source and dispersal mechanism of harmful allochthonous and autochthonous pathogens [9, 78]; however, to date, limited direct evidence of pathogenicity or virulence has been demonstrated for the marine plastisphere [11]. Using our multi-omic workflow and combined protein search strategy, we revealed the presence and activity of a range of genera which include potentially pathogenic species. Among them were those of greater clinical concern, such as *Escherichia*, *Neisseria*, *Mycobacterium*, and *Mycoplasma*, in addition to genera whereby many marine species are benign and in fact play important roles in biogeochemical cycling and bioremediation, including *Streptomyces*, *Pseudomonas*, and *Vibrio.* Virulence factors and mechanisms of antimicrobial resistance were identified within our plastisphere samples using both the comprehensive multi-omics and targeted protein search strategies. Some of the expressed virulence mechanisms were suggestive of antagonistic interactions between plastisphere community members [79], such as lipase, toxins, and iron acquisition, and these proteins were taxonomically identified as *Pseudoalteromonas*, *Streptomyces*, and *Lysobacter*, respectively. Interestingly, the expression of *Streptomyces* acyl-homoserine lactone synthase, involved in quorum sensing, suggests possible regulation of the observed toxin production [80]. Other expressed virulence mechanisms identified using VFDB, including *Francisella* sp. elongation factor Tu and *Pseudomonas aeruginosa* B-type flagellin, are well-known attachment strategies facilitating adhesion to components of host extracellular matrix [52] and cellular invasion [81]. Their expression within the plastisphere could represent general functions of biofilm formation [82], and indeed several genera (*Pseudoalteromonas*, *Sulfitobacter*, *Marinobacter*, *Colwellia*, *Psychrobacter*, and *Paracoccus*) were also expressing elongation factor Tu. Yet, confirmation herein of active potential pathogens of clinical relevance within the plastisphere, not so far identified using metaproteomics [14, 15], reflects the potential risks of human exposure to marine plastic pollution. Moreover, the identification of genes and expression of proteins that confer antimicrobial resistance to fluoroquinolones and beta-lactams, among others, in *Escherichia*, *Neisseria*, and *Mycoplasmoides*, reflects the alarming prospect that marine plastic pollution may facilitate the spread of antimicrobial resistance in the environment [83, 84]. Nonetheless, widespread confirmation of microbial virulence and pathogenicity in the plastisphere is required to truly elucidate these risks, with caution required when generalising plastisphere metabolism while functional understanding is still limited.

### Concluding remarks

To date, the functional capacities and activities of microorganisms colonizing plastic pollution are poorly resolved, and a dedicated effort is required to advance functional understanding of the marine plastisphere. To facilitate this, here we have critically assessed methodological approaches for plastisphere multi-omic and comparative metaproteomic studies and devised an optimal workflow applied to as little as 2–4 g marine plastic debris. In doing so, we have identified several areas where the use of this approach in the future could advance marine plastisphere research: Firstly, in the assessment of temperature and sunlight (in addition to other environmental factors) as key factors regulating protein expression and community metabolism to define the trophic status of the plastisphere as a feature of location. Although still an emerging research topic, this appears to play a pivotal role in the presence of hydrocarbonoclastic lineages [20, 85, 86], driving the potential for biodegradation within the plastisphere, with the success of bioremediation potentially improved through increased focus on cold-adapted metabolisms [54, 87]. Secondly, by improving the overall functional representation of the marine plastisphere, the sensitivity of techniques such as comparative metaproteomics can be continually improved through the development of specific databases which capture the diversity of peptide and protein sequences truly present in this environment [66]. Furthermore, deeper insights into plastisphere functioning may be realized through the application of data-independent acquisition mass spectrometry, which would enable access to the least abundant proteins to produce a more comprehensive view of the metaproteome for quantification [88]. In turn, shedding light on the active plastisphere organisms of broad biotechnological significance could provide new avenues of biodiscovery beyond plastic biodegradation (e.g., secondary metabolites, antimicrobials). Where plastic is utilized as a surface rather than a substrate for growth, understanding plastisphere metabolism is still of critical importance. For example, by representing potential hotspots of biogeochemical activity [89] and acting as sources and sinks of carbon and other elements within the marine environment, in addition to serving as lagrangian vectors of allochthonous and autochthonous species [90] such as pathogens. Collectively, future studies employing multi-omics and comparative metaproteomics to resolve the functional roles and activity of microorganisms inhabiting this pervasive and ubiquitous niche are strongly encouraged, to refine the broadscale impacts of plastic pollution on present and future oceans.

### Supplementary Information

**Additional file 1: Table S1.** Summary of the metaproteome and metagenome annotations. Including, taxonomic annotations (LCA score ≥80%) and functional annotations (BLASTP, Uniprot) obtained from mPies for the 16S-TaxDB, 18S-TaxDB (note taxonomy is from BLASTP results), MG-DB, and 16S-TaxDB-2nd protein search databases. The metagenome annotations are derived from DRAM and include hits to the KEGG and PFam databases. **Table S2.** Summary of the taxonomic classifications of the metagenome reads obtained using Kaiju. **Table S3.** Relative quantification and functional annotations of the expressed *Pseudoalteromonas* sp. proteins (% relative abundance) using the recovered metagenome-assembled genome as a protein search database.

**Additional file 2: Fig. S1.** Coverages of identified peptide spectra across four protein search databases for the indirect and direct extraction metaproteomes (A); corresponding number of identified proteins at a False Discovery Rate of 1% (B); number of identified peptides (C). **Fig. S2.** Significant up- / down-regulation of quantified proteins between Indirect (maroon circles) and Direct (orange circles) extraction (cut-off = 2-Fold Change, adjusted *P*-value < 0.05). **Fig. S3.** Mechanical detachment approaches and mechanical and chemical cell lysis protocols display differential plastisphere recovery and protein yields. Recovery of plastisphere cells via four detachment methods (*n* = 3; A); crystal violet biofilm assay post-detachment (*n*=3; B); total protein following mechanical cell lysis (*n*=12; C); normalised protein yields from chemical cell lysis (*n*=3; D). **Fig S4.** Microbial growth after 24 hr incubation in nutrient rich media following: mechanical detachment of plastisphere cells (*n*=3; A), and viability of chemically lysed cells (*n*=3; B). ASW = artificial seawater. Control = plastics on ice in ASW; cells = detached cells, no cell lysis. **Fig. S5.** Comparison of the annotated combined gel-free and combined gel-based metaproteomes identified shared and unique proteins and differences in the representation of functional. **Fig. S6.** Metabolic overview of the key pathways identified within the plastisphere co-assembled metagenome. **Fig. S7.** Identification of bacterial virulence factors in the plastisphere through alignment of the metagenome reads to VFDB, revealed the presence of pathogenic taxa (A) and their virulence genes (B), shown as a proportion (Relative abundance, %) of the reads aligning to VFDB. Virulence factors highlighted in red were also identified within the metaproteomic data using multiple databases (16S-TaxDB, MG-DB, 16S-TaxDB-2nd and VFDB).

**Additional file 3.**

## Data Availability

The proteomic datasets generated during the current study are available in the MassIVE repository (MSV000092214) and on Proteome Xchange (PXD043132). Sequence data are available at NCBI under BioProject PRJNA986864. Additional data are included in this article and its supplementary information files.
