# Supplementary figures and images for: Novel functional insights into the microbiome inhabiting marine plastic debris: critical considerations to counteract the challenges of thin biofilms using multi-omics and comparative metaproteomics
Source: Microbiome. 2024 Feb 22;12:36. doi: 10.1186/s40168-024-01751-x (PMC10882806; doi:10.1186/s40168-024-01751-x)

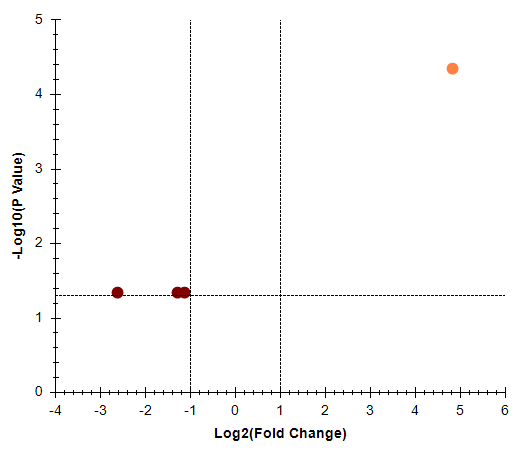

Supplement: Supplementary file 3 — Additional file 2: Fig. S1. Coverages of identified peptide spectra across four protein search databases for the indirect and direct extraction metaproteomes (A); corresponding number of identified proteins at a False Discovery Rate of 1% (B); number of identified peptides (C). Fig. S2. Significant up- / down-regulation of quantified proteins between Indirect (maroon circles) and Direct (orange circles) extraction (cut-off = 2-Fold Change, adjusted P-value < 0.05). Fig. S3. Mechanical detachment approaches and mechanical and chemical cell lysis protocols display differential plastisphere recovery and protein yields. Recovery of plastisphere cells via four detachment methods (n = 3; A); crystal violet biofilm assay post-detachment (n=3; B); total protein following mechanical cell lysis (n=12; C); normalised protein yields from chemical cell lysis (n=3; D). Fig S4. Microbial growth after 24 hr incubation in nutrient rich media following: mechanical detachment of plastisphere cells (n=3; A), and viability of chemically lysed cells (n=3; B). ASW = artificial seawater. Control = plastics on ice in ASW; cells = detached cells, no cell lysis. Fig. S5. Comparison of the annotated combined gel-free and combined gel-based metaproteomes identified shared and unique proteins and differences in the representation of functional. Fig. S6. Metabolic overview of the key pathways identified within the plastisphere co-assembled metagenome. Fig. S7. Identification of bacterial virulence factors in the plastisphere through alignment of the metagenome reads to VFDB, revealed the presence of pathogenic taxa (A) and their virulence genes (B), shown as a proportion (Relative abundance, %) of the reads aligning to VFDB. Virulence factors highlighted in red were also identified within the metaproteomic data using multiple databases (16S-TaxDB, MG-DB, 16S-TaxDB-2nd and VFDB). [file 40168_2024_1751_MOESM2_ESM.zip › Figure_S2_ESM.png]
