# Supplementary material for: Novel functional insights into the microbiome inhabiting marine plastic debris: critical considerations to counteract the challenges of thin biofilms using multi-omics and comparative metaproteomics
Source: Microbiome. 2024 Feb 22;12:36. doi: 10.1186/s40168-024-01751-x (PMC10882806; doi:10.1186/s40168-024-01751-x)
Supplement: Supplementary file 4 — Additional file 3. [file 40168_2024_1751_MOESM3_ESM.docx]

Supporting Information for:

**Novel functional insights into the microbiome inhabiting marine plastic debris: critical considerations to counteract the challenges of thin biofilms using multi-omics and comparative metaproteomics.**

Lauren F. Messer^1^, Charlotte E. Lee^1^, Ruddy Wattiez^2^, Sabine Matallana-Surget^1*^

^1^ Division of Biological and Environmental Sciences, Faculty of Natural Sciences, University of Stirling, Stirling, FK9 4LA, Scotland

^2^ Proteomic and Microbiology Department, University of Mons, Mons, 7000, Belgium

*Corresponding author: Sabine Matallana-Surget, Email: sabine.matallanasurget@stir.ac.uk, Phone: +441786 467774

# Methods

*16S and 18S rRNA gene sequencing analysis*

QIIME2 (v2022.2) [1] was used to analyse the 16S and 18S rRNA gene sequences separately, including: demultiplexing, adaptor trimming using cutadapt [2], paired-end read merging using vsearch [3], with sequence quality control (read correction, chimera and low-quality reads removal) and Amplicon Sequence Variant (ASV) generation using the Deblur workflow [4] and the SILVA 138 99% reference database [5] for the 18S rRNA data. Taxonomy was assigned to 16S and 18S rRNA ASVs separately, using a naïve-Bayes approach of the scikit learn Python package[6] with the SILVA 138 nonredundant 99% reference dataset [5]. Alpha diversity metrics were generated for rarefied 16S- and 18S-ASVs (9981 and 15961 sequences, respectively), using the QIIME2 q2-diversity core-metrics-phylogenetic plugin [7].

#### Metagenomic sequencing and analysis

Library preparation for metagenomic sequencing on the Oxford Nanopore MinION Mk1c included DNA end repair (NEBNext ONT Companion Module) and barcoding (Native Barcoding Kit 24; SQK-NBD112.24). Approximately 5fmol of the resulting library was loaded onto the ONT MinION (Mk1C; FLO-MIN106) following the standard operating procedure. Sequencing was performed for 24 hours with the following parameters established in MinKnow (v22.10.5): discard reads < 200 bp and quality scores < 7, with basecalling in real-time using Guppy (v6.3.8). This resulted in ~1.5 million reads and ~6 Gbp of metagenomic sequence. Fastq reads were trimmed using BBDuk (Min. quality score 7, each end) and normalised using BBNorm. Quality-controlled reads were combined to ensure sufficient coverage of the microbial community and taxonomically annotated using Kaiju [8], co-assembled into contiguous sequences using MetaFlye [9], setting -m to 5, and contigs were binned into metagenome-assembled genomes (MAGs) using CONCOCT [10]. Functional annotations were performed using DRAM [11], and MAG completeness and contamination was determined using CheckM [12] (v1.0.18) with taxonomy assigned using the Genome Taxonomy Database [13] (GTDBtk, v 1.7.0), leveraging the KBase interactive metagenomics environment [14]. Key metabolic processes of interest, namely plastic biodegradation (PlasticDB) [15], virulence factors (VFDB) [16], and antimicrobial resistance (CARD) [17], were explored through alignment of the reads using Minimap2 [18] with default parameters.

# ****Discussion****

#### Considerations for protein quantification using the Bradford Assay

Interestingly, although our findings suggested that direct co-extraction resulted in higher DNA and protein concentrations, this did not translate to higher peptide spectra coverages or protein identification rates within the gel-free metaproteomes across the different protein search strategies. This led to the hypothesis that direct co-extraction may increase nonprotein interference with the colorimetric Bradford Assay used for protein quantification. Although the Bradford Assay specifically binds to the amino acids arginine, lysine, and histidine, nonprotein compounds producing absorbance at 595 nm can result in protein overestimation [19,20], such as polyphenols [21], polysaccharides [22], and pharmaceutical polymers [23]. As plastisphere cell extracts comprise a mixture of extracellular polymeric substances, persistent organic pollutants, and polycyclic aromatic hydrocarbons [24], it is indeed likely that interference with the Bradford Assay occurred. Herein, we confirmed protein yields via SDS-PAGE and identified an approximately 5-fold overestimation of protein concentration based on the Bradford Assay and adjusted our sample and reagent concentrations for mass spectrometry accordingly. Yet relatively low peptide spectra coverages were still observed across our protein search strategies, reflecting the well-established challenges of analysing complex environmental samples [25,26]. Indeed, peptide spectra coverages are known to vary widely between metaproteomic studies and a concerted effort has been made in recent years to address this issue within discrete ecosystems, such as soil [26], the human gut [27], and hydrothermal vents [28]. Low peptide spectra matches likely reflected the poor representation of the active taxa and their proteins in public repositories, such as Eukaryotes and uncultivated Bacteria and Archaea, as reported previously [29], and insufficient sequencing depth of the corresponding metagenome to capture the complexity of the entire microbial community [25]. Consistency between Bradford Assay quantification, relative protein yields observed using SDS-PAGE, and protein identification rates, is seldom reported in the literature. In future, we highly recommend the use of SDS-PAGE to confirm protein yields from complex plastisphere samples, even when ultimately using a gel-free protein fractionation approach.

# References

1. Bolyen E, Rideout JR, Dillon MR, Bokulich NA, Abnet CC, Al-Ghalith GA, et al. Reproducible, interactive, scalable and extensible microbiome data science using QIIME 2. Nat Biotechnol. 2019;37:852–7.

2. Martin M. Cutadapt removes adapter sequences from high-throughput sequencing reads. EMBnet.journal. 2011;17:10–2.

3. Rognes T, Flouri T, Nichols B, Quince C, Mahé F. VSEARCH: a versatile open source tool for metagenomics. PeerJ. 2016;4:e2584.

4. Amir A, McDonald D, Navas-Molina JA, Kopylova E, Morton JT, Zech Xu Z, et al. Deblur Rapidly Resolves Single-Nucleotide Community Sequence Patterns. mSystems. 2017;2. Available from: http://dx.doi.org/10.1128/mSystems.00191-16

5. Quast C, Pruesse E, Yilmaz P, Gerken J, Schweer T, Yarza P, et al. The SILVA ribosomal RNA gene database project: improved data processing and web-based tools. Nucleic Acids Res. 2013;41:D590-6.

6. Bokulich NA, Kaehler BD, Rideout JR, Dillon M, Bolyen E, Knight R, et al. Optimizing taxonomic classification of marker-gene amplicon sequences with QIIME 2’s q2-feature-classifier plugin. Microbiome. 2018;6:90.

7. Estaki M, Jiang L, Bokulich NA, McDonald D, González A, Kosciolek T, et al. QIIME 2 enables comprehensive end-to-end analysis of diverse microbiome data and comparative studies with publicly available data. Curr Protoc Bioinformatics. 2020;70:e100.

8. Menzel P, Ng KL, Krogh A. Fast and sensitive taxonomic classification for metagenomics with Kaiju. Nat Commun. 2016;7:11257.

9. Kolmogorov M, Bickhart DM, Behsaz B, Gurevich A, Rayko M, Shin SB, et al. metaFlye: scalable long-read metagenome assembly using repeat graphs. Nat Methods. 2020;17:1103–10.

10. Alneberg J, Bjarnason BS, de Bruijn I, Schirmer M, Quick J, Ijaz UZ, et al. Binning metagenomic contigs by coverage and composition. Nat Methods. 2014;11:1144–6.

11. Shaffer M, Borton MA, McGivern BB, Zayed AA, La Rosa SL, Solden LM, et al. DRAM for distilling microbial metabolism to automate the curation of microbiome function. Nucleic Acids Res. 2020;48:8883–900.

12. Parks D.H., Imelfort M., Skennerton C.T., Hugenholtz P., Tyson G.W. CheckM: assessing the quality of microbial genomes recovered from isolates, single cells, and metagenomes. Genome Research. 2015;25:1043–55.

13. Chaumeil P-A, Mussig AJ, Hugenholtz P, Parks DH. GTDB-Tk: a toolkit to classify genomes with the Genome Taxonomy Database. Bioinformatics. 2019;36:1925–7.

14. Arkin AP, Cottingham RW, Henry CS, Harris NL, Stevens RL, Maslov S, et al. KBase: The United States Department of Energy Systems Biology Knowledgebase. Nat Biotechnol. 2018;36:566–9.

15. Gambarini V, Pantos O, Kingsbury JM, Weaver L, Handley KM, Lear G. PlasticDB: a database of microorganisms and proteins linked to plastic biodegradation. Database. 2022;2022. Available from: http://dx.doi.org/10.1093/database/baac008

16. Liu B, Zheng D, Jin Q, Chen L, Yang J. VFDB 2019: a comparative pathogenomic platform with an interactive web interface. Nucleic Acids Res. 2019;47:D687–92.

17. Alcock BP, Raphenya AR, Lau TTY, Tsang KK, Bouchard M, Edalatmand A, et al. CARD 2020: antibiotic resistome surveillance with the comprehensive antibiotic resistance database. Nucleic Acids Res. 2020;48:D517–25.

18. Sadasivan H, Maric M, Dawson E, Iyer V, Israeli J, Narayanasamy S. Accelerating Minimap2 for accurate long read alignment on GPUs. J Biotechnol Biomed. 2023;06. Available from: http://dx.doi.org/10.26502/jbb.2642-91280067

19. Compton SJ, Jones CG. Mechanism of Dye Response and Interference in the Bradford Protein Assay. Analytical Biochemistry. 1985;369–74.

20. Lu T-S, Yiao S-Y, Lim K, Jensen RV, Hsiao L-L. Interpretation of biological and mechanical variations between the Lowry versus Bradford method for protein quantification. N Am J Med Sci. 2010;2:325–8.

21. Redmile-Gordon MA, Armenise E, White RP, Hirsch PR, Goulding KWT. A comparison of two colorimetric assays, based upon Lowry and Bradford techniques, to estimate total protein in soil extracts. Soil Biol Biochem. 2013;67:166–73.

22. Banik SP, Pal S, Ghorai S, Chowdhury S, Khowala S. Interference of sugars in the Coomassie Blue G dye binding assay of proteins. Anal Biochem. 2009;386:113–5.

23. Carlsson N, Borde A, Wölfel S, Kerman B, Larsson A. Quantification of protein concentration by the Bradford method in the presence of pharmaceutical polymers. Anal Biochem. 2011;411:116–21.

24. Santana-Viera S, Montesdeoca-Esponda S, Guedes-Alonso R, Sosa-Ferrera Z, Santana-Rodríguez JJ. Organic pollutants adsorbed on microplastics: Analytical methodologies and occurrence in oceans. Trends in Environmental Analytical Chemistry. 2021;29:e00114.

25. Saito MA, Bertrand EM, Duffy ME, Gaylord DA, Held NA, Hervey WJ 4th, et al. Progress and Challenges in Ocean Metaproteomics and Proposed Best Practices for Data Sharing. J Proteome Res. 2019;18:1461–76.

26. Jouffret V, Miotello G, Culotta K, Ayrault S, Pible O, Armengaud J. Increasing the power of interpretation for soil metaproteomics data. Microbiome. 2021;9:195.

27. Stamboulian M, Li S, Ye Y. Using high-abundance proteins as guides for fast and effective peptide/protein identification from human gut metaproteomic data. Microbiome. 2021;9:80.

28. Chang Y, Fan Q, Hou J, Zhang Y, Li J. A community-supported metaproteomic pipeline for improving peptide identifications in hydrothermal vent microbiota. Brief Bioinform. 2021;22. Available from: http://dx.doi.org/10.1093/bib/bbab052

29. Oberbeckmann S, Bartosik D, Huang S, Werner J, Hirschfeld C, Wibberg D, et al. Genomic and proteomic profiles of biofilms on microplastics are decoupled from artificial surface properties. Environ Microbiol. 2021;23:3099–115.
